# Exogenous expression of an allatotropin-related peptide receptor increased the membrane excitability in *Aplysia* neurons

**DOI:** 10.1186/s13041-022-00929-4

**Published:** 2022-05-09

**Authors:** Guo Zhang, Shi-Qi Guo, Si-Yuan Yin, Wang-Ding Yuan, Ping Chen, Ji-il Kim, Hui-Ying Wang, Hai-Bo Zhou, Abraham J. Susswein, Bong-Kiun Kaang, Jian Jing

**Affiliations:** 1 State Key Laboratory of Pharmaceutical Biotechnology, Institute for Brain Sciences, Advanced Institute for Life Sciences, School of Life Sciences, Nanjing University, Nanjing, 210023 Jiangsu China; 2 School of Biological Sciences, Seoul National University, 1, Gwanak-ro, Gwanak-gu, Seoul, 08826 Korea; 3 School of Electronic Science and Engineering, Nanjing University, Nanjing, 210023 Jiangsu China; 4 Peng Cheng Laboratory, Shenzhen, 518000 China; 5 The Mina and Everard Goodman Faculty of Life Sciences, Bar Ilan University, 52900 Ramat Gan, Israel; 6 Department of Neuroscience, Icahn School of Medicine at Mount Sinai, New York, NY 10029 USA

**Keywords:** Neuropeptide, G-protein coupled receptors, *Aplysia*, Plasmid microinjection, Electrophysiology, Neuronal excitability

## Abstract

Neuropeptides act mostly on a class of G-protein coupled receptors, and play a fundamental role in the functions of neural circuits underlying behaviors. However, physiological functions of some neuropeptide receptors are poorly understood. Here, we used the molluscan model system *Aplysia* and microinjected the exogenous neuropeptide receptor apATRPR (*Aplysia* allatotropin-related peptide receptor) with an expression vector (pNEX3) into *Aplysia* neurons that did not express the receptor endogenously. Physiological experiments demonstrated that apATRPR could mediate the excitability increase induced by its ligand, apATRP (*Aplysia* allatotropin-related peptide), in the *Aplysia* neurons that now express the receptor. This study provides a definitive evidence for a physiological function of a neuropeptide receptor in molluscan animals.

**Supplementary Information:**

The online version contains supplementary material available at 10.1186/s13041-022-00929-4.

Neuropeptides, the most diverse class of neurotransmitters/neuromodulators, largely act on G-protein coupled receptors (GPCRs). Diversity arises in part from the possibility that a single neuropeptide precursor can generate multiple forms of active peptides, and a peptide can act on multiple GPCRs, which in turn might function through different signaling pathways [1]. Relatively simple model systems such as *Aplysia* are often used to study neuropeptide signaling. Earlier studies in model systems have focused on identifying neuropeptides and their bioactivity [2, 3]. Recently, growing genetic information has facilitated studying both neuropeptides and their receptors [4], e.g., expressing putative GPCRs in a cell line, and then testing activity of potential ligands on the receptors. In such systems, both receptor expression in the CNS and the physiological and/or circuit activity of the ligands are demonstrated. A match between the receptor activity of the ligands in the cell line and their physiological activity in the CNS is evidence that the receptor functions in the CNS. However, given that a peptide might act on multiple receptors, it is also necessary to demonstrate that the identified GPCR actually initiates the proper physiological activity in native neurons. Here, we used an expression vector [5, 6] to develop a method that expresses a peptide GPCR in native *Aplysia* neurons and examine whether the GPCR shows a physiological activity. Our research utilizes *Aplysia* allatotropin-related peptide (apATRP) [2] and its receptor apATRPR [4] as an example.

The neuropeptide allatotropin was first found in tissues of corpora allata in the insect *Manduca sexta* [7] and subsequently allatotropin-related peptides were characterized in Arthropoda, Annelida and Mollusca with various functions in different behaviors, including feeding. The allatotropin receptor was originally characterized in *Bombyx mori* [8], followed by identification of other insect allatotropin receptors, e.g., in *Manduca sexta* [9]. Additionally, two allatotropin receptors in the annelid *Platynereis* [10] and one in *Aplysia* [4] were characterized. Interestingly, phylogenetic analyses have shown that protostome allatotropin and deuterostome orexin signaling systems are orthologous [11].

In *Aplysia*, apATRP (GFRLNSASRVAHGY-NH2) acts on the feeding motor circuit to enhance motor neuron B61/62 excitability. This increases B61/62 firing frequency, thereby compensating for the short duration of B61/62 bursts during feeding motor programs elicited by an apATRP-positive command neuron [2]. B61/62 firing frequency also increases after learning that food is inedible [12]. Recently, several ligands, including apATRP, were found to activate apATRPR in CHO-K1 cells transiently transfected with apATRPR. Importantly, the pattern of activations of these ligands in the cell line matches their actions on B61/62 excitability, suggesting that apATRPR likely functions in the *Aplysia* CNS [4]. However, it is unknown whether apATRPR mediates the excitability increase in native *Aplysia* neurons, given that there might be multiple apATRP receptors in *Aplysia*. Here, we sought to determine whether apATRP is sufficient to mediate the ligand effect on native neurons by evaluating the ability of apATRP to activate apATRPR in *Aplysia* neurons that do not endogenously express apATRPR. To express apATRPR in neurons, we used a plasmid vector, pNEX3, which is an effective method to express exogenous proteins in cultured *Aplysia* neurons [5, 6]. Thus, we constructed the recombinant plasmid pNEX3-apATRPR (Additional file 1).

To demonstrate that apATRPR might function as an endogenous receptor of apATRP, we first sought to find a target neuron that did not natively express apATRPR in the buccal ganglia. We selected a larger neuron, B8 (~ 150 μm), and examined B8 excitability changes in response to apATRP. apATRP increased B8 excitability (Fig. 1a, b), suggesting that B8 might contain a receptor(s) for apATRP. Therefore, B8 neurons were excluded. After testing several additional neurons, we found that another large neuron B1/B2 (~ 210 μm) did not respond to apATRP (Fig. 1c, d), and it was used as the target neuron.

**Fig. 1:**
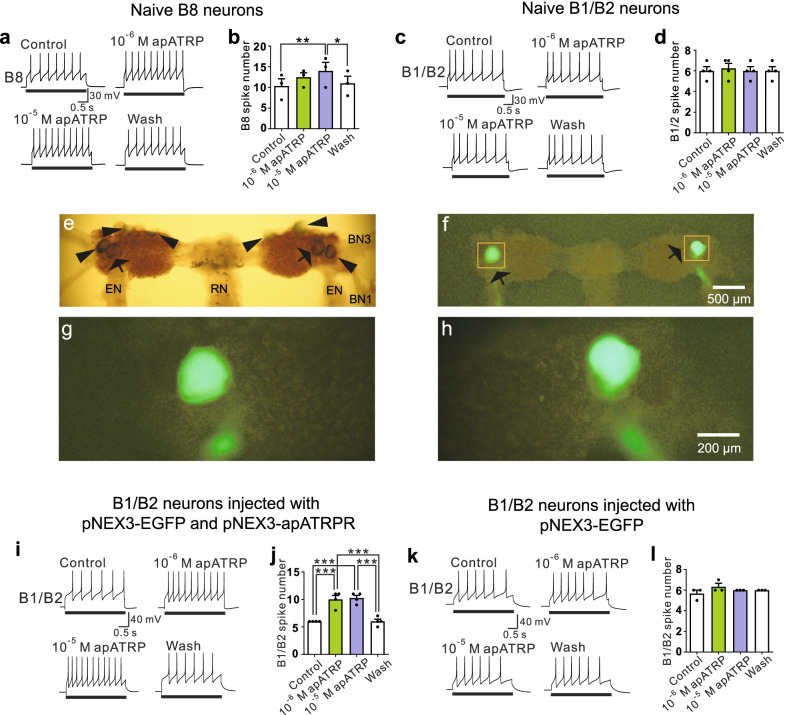
apATRP increased the excitability of B1/B2 neurons that were exogenously expressed with the receptor apATRPR. **a**, **b** apATRP increased B8 excitability at 10^–5^ M but not at 10^–6^ M (F(3, 6) = 14.89, p < 0.01, n = 3 individual neurons from three preparations). Bonferroni post hoc tests: *p < 0.05, **p < 0.01. Error bars, SE. **c**, **d** apATRP had no significant effect on B1/B2 excitability (F(3, 9) = 1.00, p > 0.05, n = 4 individual neurons from four preparations). Error bars, SE. **e** The caudal surface of a buccal ganglion viewed with a regular light source. B1/B2 and other neurons on the left side were microinjected with plasmids pNEX3-apATRPR and pNEX3-EGFP, and B1/B2 and other neurons on the right side were microinjected with only plasmid pNEX3-EGFP. Arrows indicate the neurons injected with plasmids and expressed the EGFP protein (showed bright fluorescence under a fluorescence microscope, see panel **f**). Arrowheads indicate the neurons injected with plasmids but did not express the EGFP protein. **f** B1/B2 neurons on both sides showed bright fluorescence (green, arrows) under a fluorescence microscope. The left B1/B2 neuron expressed apATRPR and EGFP, and the right B1/B2 neuron expressed EGFP. Other neurons marked with arrowheads in (**e**) did not express injected genes. **g**, **h** A magnified view of the injected neurons in (**f**) showing left B1/B2 neuron (**g**) and right B1/B2 neuron (**h**). Scale bar in **f**: 500 μm (scale bar in **f** is for **e** and **f**); Scale bar in **h**: 200 μm (scale bar in **h** is for **g** and **h**). **i**, **j** At 10^–6^ M and 10^–5^ M, apATRP increased B1/B2 excitability (F(3, 9) = 44.84, p < 0.0001, n = 4 individual neurons from three preparations), which expressed the receptor apATRPR. Bonferroni post hoc tests: ***p < 0.001. Error bars, SE. **k**, **l** apATRP had no significant effects on B1/B2 neurons that do not express the receptor apATRPR (F(3, 6) = 1.60, p > 0.05, n = 3 individual neurons from three preparations). Bars in **a**, **c**, **i** and **k** denote current injections. Control groups and wash groups in **a**, **c**, **i**, **k** were perfused with high divalent saline only, whereas the experimental groups in **a**, **c**, **i**, **k** were perfused with the neuropeptide apATRP dissolved in high divalent saline

In each hemi-ganglion of the buccal ganglion, there are one B1 and one B2 neuron. Thus, there are four B1/B2 neurons on both sides of the buccal ganglion. We set up two groups: the plasmid pNEX3-EGFP mixture with fast green microinjected into B1/B2 neurons as the control group, and the plasmid pNEX3-EGFP and pNEX3-apATRPR mixture with fast green microinjected into the contralateral B1/B2 neurons as the experimental group. Visualizing fast green with a regular light source confirmed that the plasmid injection was successful (Fig. 1e). After injection, we placed the buccal ganglion into cell culture until we observed that B1/B2 neurons exhibited green fluorescence (Fig. 1f–h), which took 1 to 3 days. Observing green fluorescence confirmed that neurons had expressed the EGFP in the control group and had co-expressed the apATRPR and EGFP in the experimental group.

We next perfused apATRP into the recording dish and tested B1/B2 excitability. The results showed that B1/B2 excitability in the experimental group was enhanced (Fig. 1i, j), and B1/B2 excitability in the control group showed no significant changes (Fig. 1k, l). This finding indicated that the neuropeptide receptor, apATRPR, could mediate the excitability increase in native *Aplysia* neurons in response to its ligand, apATRP.

In this work, we have characterized physiological functions of a neuropeptide receptor, apATRPR, expressed in *Aplysia* neurons. Our study provides a definitive evidence that apATRPR indeed mediates excitability increase in a neuron that does not express apATRPR, indicating that the neuropeptide receptor, apATRPR, is sufficient to mediate an excitability increase to its ligand, apATRP, in *Aplysia* neurons. In terms of molecular mechanisms underlying the excitability increase, we speculate that, similar to insects [9], through Gαs, apATRPR could increase cAMP, which could in turn act either to close K channels through PKA [13] or to activate cAMP-gated Na channels through a PKA-independent pathway (Additional file 1).

Taken together with earlier work showing that pNEXδ or pNEX3 can express GPCRs for glutamate, octopamine and serotonin [13–15], pNEX, including pNEXδ and pNEX3, proves to be an effective plasmid to express GPCRs for both small molecule transmitters and neuropeptides in *Aplysia* neurons. We expect that such a procedure could be readily applied to demonstrate physiological functions of neuropeptide receptors in native neurons in model systems with reasonably large identifiable neurons, such as other molluscs, annelids and possibly some arthropods. Notably, compared with invertebrate organisms such as *C. elegans* and *Drosophila*, life spans of molluscs and annelids are relatively long and life cycles are complex, making it difficult to use transgenes to manipulate gene expression. Consequently, the procedure described in this paper should be particularly useful in these animals to study functions of genes in native neurons.

## Supplementary Information

**Additional file 1. **Containing Additional discussion, detailed Material and Methods, statistics and 2 additional figures.

## Data Availability

All data generated or analyzed during this study are included in this published article and its additional information file.

## Abbreviations

EN: Esophageal nerve
BN1: Buccal nerve 1
BN3: Buccal nerve 3
pNEX: Plasmid for neuronal expression
RN: Radular nerve

## References

[1] Abid MSR, Mousavi S, Checco JW (2021). Identifying receptors for neuropeptides and peptide hormones: challenges and recent progress. ACS Chem Biol.

[2] Jing J, Sweedler JV, Cropper EC, Alexeeva V, Park JH, Romanova EV, Xie F, Dembrow NC, Ludwar BC, Weiss KR, Vilim FS (2010). Feedforward compensation mediated by the central and peripheral actions of a single neuropeptide discovered using representational difference analysis. J Neurosci.

[3] Zhang G, Vilim FS, Liu DD, Romanova EV, Yu K, Yuan WD, Xiao H, Hummon AB, Chen TT, Alexeeva V, Yin SY, Chen SA, Cropper EC, Sweedler JV, Weiss KR, Jing J (2017). Discovery of leucokinin-like neuropeptides that modulate a specific parameter of feeding motor programs in the molluscan model. Aplysia. J Biol Chem..

[4] Checco JW, Zhang G, Yuan WD, Le ZW, Jing J, Sweedler JV (2018). *Aplysia* allatotropin-related peptide and its newly identified d-amino acid-containing epimer both activate a receptor and a neuronal target. J Biol Chem.

[5] Kaang BK, Pfaffinger PJ, Grant SG, Kandel ER, Furukawa Y (1992). Overexpression of an *Aplysia* shaker K+ channel gene modifies the electrical properties and synaptic efficacy of identified *Aplysia* neurons. Proc Natl Acad Sci USA.

[6] Kaang BK (1996). Parameters influencing ectopic gene expression in *Aplysia* neurons. Neurosci Lett.

[7] Kataoka H, Toschi A, Li JP, Carney RL, Schooley DA, Kramer SJ (1989). Identification of an allatotropin from adult manduca sexta. Science.

[8] Yamanaka N, Yamamoto S, Zitnan D, Watanabe K, Kawada T, Satake H, Kaneko Y, Hiruma K, Tanaka Y, Shinoda T, Kataoka H (2008). Neuropeptide receptor transcriptome reveals unidentified neuroendocrine pathways. PLoS ONE.

[9] Horodyski FM, Verlinden H, Filkin N, Vandersmissen HP, Fleury C, Reynolds SE, Kai ZP, Broeck JV (2011). Isolation and functional characterization of an allatotropin receptor from *Manduca sexta*. Insect Biochem Mol Biol.

[10] Bauknecht P, Jekely G (2015). Large-scale combinatorial deorphanization of *Platynereis* neuropeptide GPCRs. Cell Rep.

[11] Jekely G (2013). Global view of the evolution and diversity of metazoan neuropeptide signaling. Proc Natl Acad Sci USA.

[12] Tam S, Hurwit I, Chiel HJ, Susswein AJ (2020). Multiple local synaptic modifications at specific sensorimotor connections after learning are associated with behavioral adaptations that are components of a global response change. J Neurosci.

[13] Lee YS, Choi SL, Lee SH, Kim H, Park H, Lee N, Lee SH, Chae YS, Jang DJ, Kandel ER, Kaang BK (2009). Identification of a serotonin receptor coupled to adenylyl cyclase involved in learning-related heterosynaptic facilitation in *Aplysia*. Proc Natl Acad Sci USA.

[14] Whim MD, Kaczmarek LK (1998). Expression of a foreign G-protein coupled receptor modulates the excitability of the peptidergic bag cell neurons of Aplysia. Neurosci Lett.

[15] Chang DJ, Lim CS, Lee JA, Kaang BK (2003). Synaptic facilitation by ectopic octopamine and 5-HT receptors in *Aplysia*. Brain Res Bull.

