# Supplementary material for: Exogenous expression of an allatotropin-related peptide receptor increased the membrane excitability in Aplysia neurons
Source: Mol Brain. 2022 May 9;15:42. doi: 10.1186/s13041-022-00929-4 (PMC9082908; doi:10.1186/s13041-022-00929-4)

**Additional file 1**

**Discussion**

In this work, we demonstrated that apATRPR could mediate the excitability increase in B1/B2 that do not express apATRPR endogenously. Future work is needed to determine the molecular mechanisms underlying the excitability increase. We could, however, speculate possible mechanisms based on previous studies. In insects [1-3], allatotropin receptors have been shown to increase both Ca and cAMP in cell lines expressing the receptors. Ca and cAMP are the second messengers from Gαq and Gαs pathways respectively [4]. In *Aplysia*, apATRPR acts on Gαq pathway [5], and, similar to insects, might also act on Gαs pathway to increase cAMP. Moreover, previous work in molluscs has shown that cAMP could increase membrane excitability in two ways. One is through protein kinase A (PKA) that ultimately acts to close K channels in *Aplysia* [6-10]. Second is through a PKA-independent pathway, where cAMP directly activates cAMP-gated Na channels, as has been demonstrated in *Pleurobranchaea* [11] and in *Aplysia* [12]. The above discussion could be used to guide future studies.

**Materials and Methods**

**Construction of plasmids**

Initially, pNEX was constructed with the AK01a gene (shaker K^+^ channel), and microinjected into cultured *Aplysia* neurons, which demonstrated that the AK01a channel could modulate the firing of the injected neuron and regulate synaptic interactions [13]. Subsequent studies used pNEX to overexpress the target proteins and elucidated molecular mechanisms of synaptic plasticity. pNEX has two members, pNEXδ [14] or pNEX3 [15]. There are eight more enhancers in pNEX3 than pNEXδ, making pNEX3 more effective in expressing a gene [15]. We chose to use pNEX3. Previously, plasmid vectors expressing the pNEX3 gene were constructed in *Aplysia* neurons [14,15], and overexpressed proteins in a specific neuron [14,16,17]. The plasmid pNEX3-EGFP was derived from the earlier work [18], and the plasmid pcDNA3.1-apATRPR was a gift from Dr. Checco at the University of Illinois. To generate the expression plasmid pNEX3- apATRPR, the apATRPR gene was ligated with the vector pNEX3. First, the EGFP gene was digested from the BamHI-KpnI restriction fragment of pNEX3 and separated by agarose gel electrophoresis. Second, the apATRPR gene was added to the restriction sites of BamHI and KpnI at the 5’ and 3’ ends (forward primer: CGCGGATCCATGGGGTCGAACGATACATTC; reverse primer: GGGGTACCTCAGATGCTGGCGAGAGTGACCTC), respectively, by performing polymerase chain reaction (PCR) with the pcDNA3.1-apATRPR plasmid as the template. Then, the target gene, apATRPR, and the vector, pNEX3, were ligated using T4 DNA ligase. All plasmid DNAs used in microinjection were prepared by a standard maxi-prep procedure using an EndoFree Maxi Plasmid Kit.

**Electrophysiology**

*Aplysia californica* (100−300 g) were purchased from Marinus Scientific (Long Beach, CA). *Aplysia* are hermaphroditic (i.e., each animal has functioning male and female reproductive organs). Animals were kept in an aquarium containing aerated and filtered artificial seawater (Instant Ocean, Aquarium Systems Inc., Mentor, OH) at 14−16 °C. The animal room was equipped with a 24 h light−dark cycle with a light period from 6:00 am to 6:00 pm. Prior to dissection, animals were anesthetized by injection of isotonic 333 mM MgCl_2_ (approximately 50% of body weight) into the body cavity. All reagents were purchased from Sigma–Aldrich (St. Louis, MO) unless otherwise indicated. apATRP was synthesized by ChinaPeptides Co., Ltd (Additional file 1).

Electrophysiological techniques were utilized as described previously [19,20]. Briefly, ganglia were desheathed, transferred to a recording chamber containing 1.5 mL of artificial seawater (ASW, 460 mM NaCl, 10 mM KCl, 11 mM CaCl_2_, 55 mM MgCl_2_, and 10 mM HEPES, pH 7.6), continuously perfused at 0.3 mL/min, and maintained at 14−17 °C. Physiological experiments on neuronal excitability were performed in highly divalent (HiDi) saline (368 mM NaCl, 8 mM KCl, 13.8 mM CaCl_2_, 115 mM MgCl_2_, and 10 mM HEPES, pH 7.6), which increases the spiking threshold of neurons and therefore curtails polysynaptic influences. Intracellular recordings were obtained using 5−10 MΩ sharp microelectrodes filled with an electrolyte (0.6 M K_2_SO_4_ plus 60 mM KCl). The average resting membrane potentials of B8 and B1/B2 neurons were -53 ± 2.5 mV (n = 3) and -47 ± 6.9 mV (n = 6) respectively. The average action potentials of B8 and B1/B2 neurons were 50 ± 4.5 mV (n = 3) and 55 ± 5 mV (n = 6) respectively. Grass S88 stimulator was used to provide timing signals for intracellular stimulation. Positive current pulses lasting 3 seconds were used to test excitability of single neurons, with the stimulus interval being 30 seconds. Electrophysiological recordings were digitized online using AxoScope (Molecular Devices, Sunnyvale, CA) and plotted by CorelDraw (Corel Corporation, Ottawa, ON, Canada).

**Microinjection of plasmids**

For neurons in the *Aplysia* buccal ganglion that didn’t respond to apATRP, we established two groups. The control neurons were microinjected with the DNA construct pNEX3-EGFP, and the experimental neurons were microinjected with a mixture of pNEX3-EGFP and pNEX3-apATRPR. The EGFP was used as a marker of gene expression. We microinjected the plasmids by pressure injection. The pressure ranged from 20 to 30 psi. To observe the microinjected plasmid volume, the electrolyte is a mixture of 50 μl plasmids (in 1.8 mg/ml) with 50 μl 0.4% fast green buffer (fast green dissolved in 20 mM HEPES, 200 mM KCl, pH = 7.37) to inject neurons. We stopped injecting when the plasmid volume expanded ~ 1/3 of the volume of the injected neuron and the neuron could be seen to turn green due to fast green (see Fig. 1f). In the experimental group, if neurons had green fluorescence under an Olympus fluorescence microscope (see Fig. 1g), we assumed that these neurons expressed both EGFP and apATRPR.

**Culture of *Aplysia* neurons and detection of plasmid expression**

After injection, the buccal ganglia were cultured at 18 °C. The culture medium was made up of an aliquot of *Aplysia* hemolymph and L15 at 1:1 by volume [21]. Then, we added a 1% total volume of 50 mg/ml ampicillin sodium salt (Sigma–Aldrich: A9518-25G-9) solution and a 1% total volume of 200 mM L-glutamine (Sigma–Aldrich: V900419-100G). The culture medium was prepared freshly each time. *Aplysia* hemolymph was prepared from large live *Aplysia* (> 350 gm) using a syringe through a 0.22 μm filter, and aliquots of hemolymph were stored at -80 ℃. L-15 medium powder (Leibovitz) (Gibco: 41300039) was supplemented with the salts to make 1-liter solution (L-15 power 13.7 g, NaCl 15.4 g, D-Glucose 6.24 g, MgSO_4_ 3.15 g, KCl 0.35 g, NaHCO_3_ 0.17 g, MgCl_2_·6H_2_O 5.49 g, CaCl_2_ 1.08 g, HEPES 3.53 g, pH, 7.4-7.5; osmolarity: ~ 1000 mmol/kg). Then, 10 ml of 100 x (5 mg/ml) gentamicin sulfate salt (Sigma–Aldrich: E003632-1G) solution was added. The mixture was filter-sterilized through a 0.22 μm filter, and stored at 4℃.

During ganglia culture, the gene expression was observed using an Olympus fluorescence microscope every day. If the neurons in the control and experimental groups expressed green fluorescence, ganglia were then transferred to an electrophysiological setup to evaluate the activity of apATRP on these neurons using the procedures described in the “Electrophysiology” section.

**Data analysis**

Bar graphs were plotted with Prism (version 8, GraphPad Software, La Jolla, CA). Data are expressed as the mean ± S.E.M. All statistical tests were performed using Prism. When the data showed significant effects in ANOVA, individual comparisons were performed with Bonferroni’s correction.

**Reference**

[1] F.M. Horodyski, H. Verlinden, N. Filkin, H.P. Vandersmissen, C. Fleury, S.E. Reynolds, Z.P. Kai, J.V. Broeck, Isolation and functional characterization of an allatotropin receptor from *Manduca sexta*, Insect Biochem Mol Biol 41 (2011) 804-814.

[2] H. Verlinden, E. Lismont, M. Bil, E. Urlacher, A. Mercer, J. Vanden Broeck, R. Huybrechts, Characterisation of a functional allatotropin receptor in the bumblebee, *Bombus terrestris* (Hymenoptera, Apidae), Gen Comp Endocrinol 193 (2013) 193-200.

[3] E. Lismont, R. Vleugels, E. Marchal, L. Badisco, P. Van Wielendaele, C. Lenaerts, S. Zels, S.S. Tobe, J. Vanden Broeck, H. Verlinden, Molecular cloning and characterization of the allatotropin precursor and receptor in the desert locust, *Schistocerca gregaria*, Front Neurosci 9 (2015) 84.

[4] D. Purves, Augustine, G. J., Fitzpatrick, D., Hall, W. C., Lamantia, A. S., McNamara, J. O., & Williams, S. M. , Neuronscience, 2004.

[5] J.W. Checco, G. Zhang, W.D. Yuan, Z.W. Le, J. Jing, J.V. Sweedler, *Aplysia* allatotropin-related peptide and its newly identified d-amino acid-containing epimer both activate a receptor and a neuronal target, The Journal of biological chemistry 293 (2018) 16862-16873.

[6] D.A. Baxter, J.H. Byrne, Differential effects of cAMP and serotonin on membrane current, action-potential duration, and excitability in somata of pleural sensory neurons of *Aplysia*, J. Neurophysiol. 64 (1990) 978-990.

[7] B.A. Goldsmith, T.W. Abrams, cAMP modulates multiple K+ currents, increasing spike duration and excitability in *Aplysia* sensory neurons, Proc. Natl. Acad. Sci. USA 89 (1992) 11481-11485.

[8] J.H. Byrne, E.R. Kandel, Presynaptic facilitation revisited: state and time dependence, J. Neurosci. 16 (1996) 425-435.

[9] D.J. Chang, X.C. Li, Y.S. Lee, H.K. Kim, U.S. Kim, N.J. Cho, X. Lo, K.R. Weiss, E.R. Kandel, B.K. Kaang, Activation of a heterologously expressed octopamine receptor coupled only to adenylyl cyclase produces all the features of presynaptic facilitation in *Aplysia* sensory neurons, Proc Natl Acad Sci U S A 97 (2000) 1829-1834.

[10] Y.S. Lee, S.L. Choi, S.H. Lee, H. Kim, H. Park, N. Lee, S.H. Lee, Y.S. Chae, D.J. Jang, E.R. Kandel, B.K. Kaang, Identification of a serotonin receptor coupled to adenylyl cyclase involved in learning-related heterosynaptic facilitation in *Aplysia*, Proc Natl Acad Sci U S A 106 (2009) 14634-14639.

[11] L.C. Sudlow, R.-C. Huang, D.J. Green, R. Gillette, cAMP-activated Na+ current of molluscan neurons is resistant to kinase inhibitors and is gated by cAMP in the isolated patch, J. Neurosci. 13 (1993) 5188-5193.

[12] M.H. Perkins, K.R. Weiss, E.C. Cropper, Persistent effects of cyclic adenosine monophosphate are directly responsible for maintaining a neural network state, Sci Rep 9 (2019) 9058.

[13] B.K. Kaang, P.J. Pfaffinger, S.G.N. Grant, E.R. Kandel, Y. Furukawa, Overexpression of an *Aplysia* shaker K^+^ channel gene modifies the electrical properties and synaptic efficacy of identified *Aplysia* neurons, Proc. Natl. Acad. Sci. USA 89 (1992) 1133-1137.

[14] B.K. Kaang, P.J. Pfaffinger, S.G. Grant, E.R. Kandel, Y. Furukawa, Overexpression of an *Aplysia* shaker K+ channel gene modifies the electrical properties and synaptic efficacy of identified *Aplysia* neurons, Proc Natl Acad Sci U S A 89 (1992) 1133-1137. 10.1073/pnas.89.3.1133.

[15] B.K. Kaang, Parameters influencing ectopic gene expression in *Aplysia* neurons, Neurosci Lett 221 (1996) 29-32. 10.1016/s0304-3940(96)13279-1.

[16] T. Nagahama, T. Suzuki, S. Yoshikawa, M. Iseki, Functional transplant of photoactivated adenylyl cyclase (PAC) into *Aplysia* sensory neurons, Neurosci Res 59 (2007) 81-88. 10.1016/j.neures.2007.05.015.

[17] J. Dyer, W.S. Sossin, Characterization of the role of eIF4G in stimulating cap- and IRES-dependent translation in *Aplysia* neurons, PLoS One 8 (2013) e74085. 10.1371/journal.pone.0074085.

[18] J.A. Lee, C.S. Lim, S.H. Lee, H. Kim, N. Nukina, B.K. Kaang, Aggregate formation and the impairment of long-term synaptic facilitation by ectopic expression of mutant huntingtin in *Aplysia* neurons, J Neurochem 85 (2003) 160-169. 10.1046/j.1471-4159.2003.01650.x.

[19] L. Bai, I. Livnat, E.V. Romanova, V. Alexeeva, P.M. Yau, F.S. Vilim, K.R. Weiss, J. Jing, J.V. Sweedler, Characterization of GdFFD, a D-amino acid-containing neuropeptide that functions as an extrinsic modulator of the *Aplysia* feeding circuit, J Biol Chem 288 (2013) 32837-32851. DOI 10.1074/jbc.M113.486670.

[20] J. Jing, V. Alexeeva, S.A. Chen, K. Yu, M.R. Due, L.N. Tan, T.T. Chen, D.D. Liu, E.C. Cropper, F.S. Vilim, K.R. Weiss, Functional characterization of a vesicular glutamate transporter in an interneuron that makes excitatory and inhibitory synaptic connections in a molluscan neural circuit, J Neurosci 35 (2015) 9137-9149. 10.1523/JNEUROSCI.0180-15.2015.

[21] M. Fejtl, J. Gyori, D.O. Carpenter, Mercuric(II) chloride modulates single-channel properties of carbachol-activated Cl- channels in cultured neurons of *Aplysia californica*, Cell Mol Neurobiol 14 (1994) 665-674. 10.1007/BF02088675.

**Figure S1. Complete gels for verification of pNEX3-apATRPR and apATRPR genes**. Left panels: complete gels. The lanes in the red rectangle are our target genes and the marker lane. The other lanes are unrelated genes. Right panels: Gel electrophoresis of the plasmid pNEX3-apATRPR (approximately 4274 bp) and apATRPR gene (approximately 1215 bp) digested from the BamHI-KpnI restriction fragment of pNEX3 (approximately 3059 bp). apATRPR gene has been verified by sequencing. Lane 1: plasmid pNEX3-apATRPR; lane 2: apATRPR gene (the lower band) and pNEX3 vector (the upper band); M: marker.


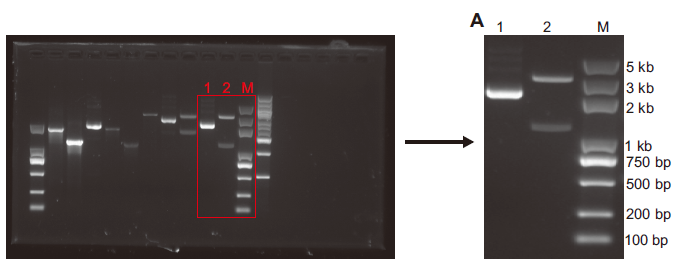


**Figure S2. Peptide synthesis and quality information from commercial companies**. COA is on part 1, HPLC analysis report on part 2, MS analysis report on part 3.


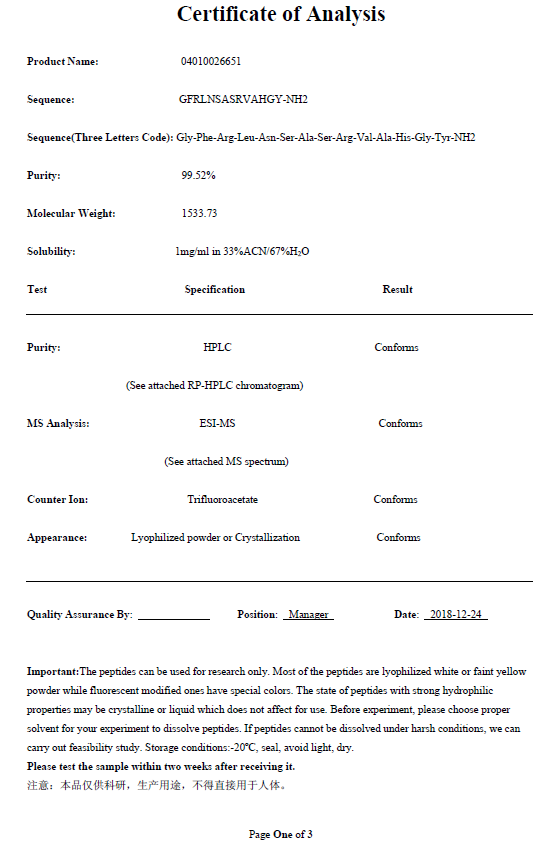
Part 1

Part 2


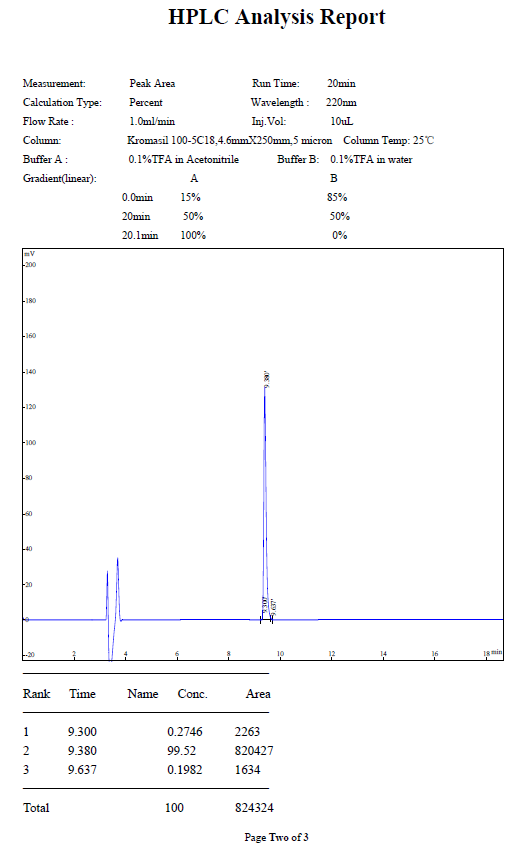


Part 3
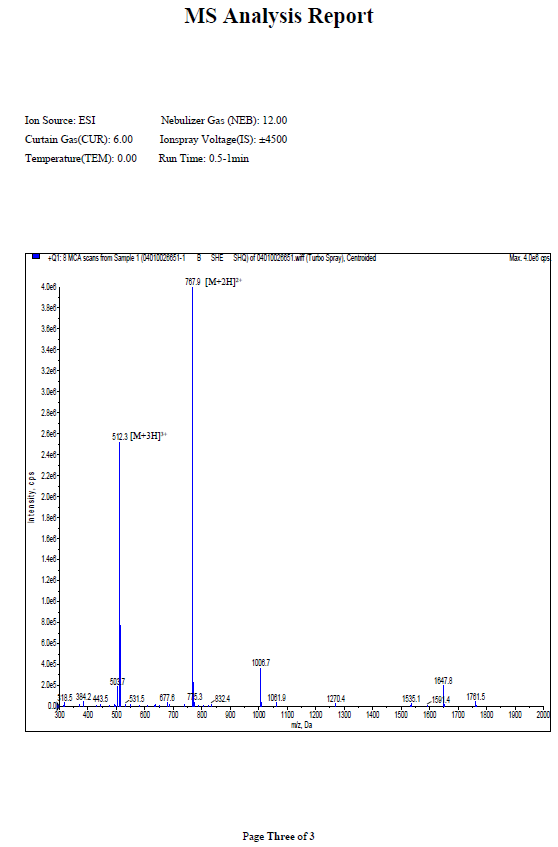

Supplement: Supplementary file 1 — Additional file 1. Containing Additional discussion, detailed Material and Methods, statistics and 2 additional figures. [file 13041_2022_929_MOESM1_ESM.docx]
